# New Insights Into Energy Substrate Utilization and Metabolic Remodeling in Cardiac Physiological Adaption

**DOI:** 10.3389/fphys.2022.831829

**Published:** 2022-02-25

**Authors:** Xiaomeng Shi, Hongyu Qiu

**Affiliations:** Center for Molecular and Translational Medicine, Institute of Biomedical Sciences, Georgia State University, Atlanta, GA, United States

**Keywords:** metabolism, substrate utilization, heart, physiology, cardiac disease

## Abstract

Cardiac function highly relies on sufficient energy supply. Perturbations in myocardial energy metabolism play a causative role in cardiac pathogenesis. Accumulating evidence has suggested that modifications of cardiac metabolism are also an essential part of the adaptive responses to various physiological conditions in the heart to meet specific energy needs. The review highlighted some new studies on basic myocardial energy substrate metabolism and updated recent findings regarding cardiac metabolic remodeling and their associated mechanisms under physiological conditions, including exercise and cardiac development. Studying basic metabolic profiles in the heart in these conditions can contribute to understanding the significance of metabolic regulation in the heart during physiological adaption and gaining further insights into the maladaptive metabolic changes associated with cardiac pathogenesis, thus opening up new avenues to exploring novel therapeutic strategies in cardiac diseases.

## Introduction

The heart demands high energy production and turnover to maintain its proper functioning. Cardiomyocytes navigate their metabolic activities to meet specific energy demands with each contraction cycle. Multifaceted interactions and coordinations between metabolic pathways and networks underlie the integrated metabolic homeostasis of the heart regarding ATP production, biosynthesis, signaling regulation, and control of redox state. Perturbations in myocardial energy metabolism have been considered causative for cardiac pathogenesis, eventually progressing to ventricular dysfunction, arrhythmias, and heart failure, contributing to poor prognosis and high cardiovascular mortality rates. Therefore, it is of great significance to elucidate the critical role of metabolic regulation in the heart.

Metabolic remodeling, in general concept, refers to the reconstruction of the metabolic landscape or metabolomics profiling that results in a shift toward a greater reliance on alternative fuel metabolism (van Bilsen et al., 2008), which is involved in the regulation of essential cellular functions such as the process of substrate utilization, the tricarboxylic acid (TCA) cycle, oxidative phosphorylation, and high-energy phosphate metabolism. Although numerous studies have focused on the abnormal metabolic remodeling in heart diseases, such as myocardial infarction (MI), diabetic cardiomyopathy (DCM), and heart failure (HF) (Chong et al., 2017; Chen et al., 2019; Zuurbier et al., 2020), the mechanisms and characteristics of cardiac metabolic remodeling in modulating either beneficial or detrimental cardiac functional processes remain largely unknown. On the other hand, accumulating evidence has suggested that cardiac metabolic remodeling is an essential adaptation to various physiological conditions of the heart to meet the specific energy requirements, such as during exercise and cardiac development. Under these physiological conditions, the heart can efficiently switch energy substrate utilization to provide sufficient ATP supply to the constantly varying demand of cardiac myocytes through a complex network of signaling pathways. Gaining more knowledge in this specific field will enable us to achieve a deeper understanding of the fundamental biology and metabolism in cardiac function and explore novel therapeutic strategies to slow down or reverse pathological remodeling, thus integratively promoting overall cardiac health.

In the review, we summarized continuing research progress on myocardial energy substrate utilization and also discussed a portion of recent findings regarding cardiac metabolic remodeling and the associated mechanisms under different physiological conditions, including exercise and cardiac development. Reviewing and discussing the current progress on cardiac metabolism in these conditions can contribute to better understanding the significance of metabolic regulations in the heart during physiological adaption and receiving fresh perspectives on the maladaptive metabolic changes underlying cardiac pathogenesis.

## New Progress of Metabolic Substrate Utilization in the Heart

Virtually, the selection, partition, and coordination of different substrates for energy production in the myocardium ultimately depend on the complex cardiac dynamics over time. Lately, Murashige et al. (2020) conducted a comprehensive and quantitative mapping of cardiac fuel use via measuring dynamic uptake and release of metabolites in the human heart by liquid chromatography-mass spectrometry. It was found that the heart consumed all species of free fatty acids (FFAs) and acquired the most carbons from FFAs, taking up over 70% of total carbons extracted from the circulation. The heart acquired another 15% of total carbons from ketone extraction. Acetate, which accounted for the highest percentage of short-chain FAs produced by gut bacteria, was avidly extracted by the heart, accounting for 2% of myocardial carbon uptake. Meanwhile, the heart secreted most of the essential amino acids, particularly those with greater nitrogen content. Among them, glutamine and alanine were the two most abundant amino acids secreted, with the release of the former nearly equal to glutamate uptake. In addition, histidine was identified to be the most highly secreted essential amino acid, and considering that myoglobin, a heme protein abundantly present in cardiac tissues, contained a greater histidine content than other cardiac proteins, they could be serving as a potential carbon reservoir (Murashige et al., 2020). In comparison, the failing hearts of patients with a left ventricular ejection fraction (LVEF) below 40% exhibited an almost threefold increase in ketone consumption, a twofold increase in lactate consumption, and a doubled rate of proteolysis together with suppressed FFA use, but there was no evidence of increased secretion of acylcarnitines found, which contradicted with the previous findings that associated increased plasma long-chain acylcarnitines with heart failure (Ahmad et al., 2016). Myocardial consumption of three fuels, including acetate, 3-hydroxybutyrate (3-OHB), and glutamate, was directly proportional to their circulating concentrations, which suggested that the utilization of these substrates were predominantly driven by their availability instead of relying on substantial regulations like glucose and FFAs. Furthermore, the fractional uptake of these three fuels exhibited a strong correlation with each other, which suggested that tissue perfusion was another key determinant for their fuel utilization efficiency. Unexpectedly, lactate uptake by the heart was also not proportional to its blood concentrations, which directly contradicted the previous evidence stating that myocardial lactate extraction was directly proportional to its circulating concentrations (Gertz et al., 1988). As it is known, cardiac metabolism is a vital research topic and has been extensively characterized in various models of heart diseases in the past. Thus, this review mainly highlighted some of the new progress about myocardial metabolism, focused on the newly developed methodologies and the recent discovery of metabolic substrate utilization, to improve our understanding of this appreciated topic.

### Glucose and Fatty Acids

Unlike most tissues, the heart mainly utilizes long-chain fatty acids, accounting for 60–70% of its fuel metabolism. Distinctively, glucose oxidation is less favored in the heart because the heart has very little glycogen available. It gets depleted very quickly under hypoxic conditions, plus little gluconeogenesis occurs in the heart. Metabolic remodeling involving glucose and lipid metabolism has been intensely characterized in the heart with preexisting dysfunctions, such as heart failure, myocardial infarction, and diabetic cardiomyopathy. In mature animal models of heart failure that is most commonly elicited by either pressure-overload or ischemia, the energy-deficient heart switches from fatty acid oxidation to more oxygen-efficient glucose oxidation accompanied by markedly upregulated cardiac glucose uptake (Nabben et al., 2018). Such metabolic shift is not always detrimental as higher oxygen efficiency could ameliorate ischemia in the hypoxic myocardium, but not in pressure-overloaded heart. Conversely, the diabetic heart has to depend more on fatty acid oxidation to compensate for crippled glucose oxidation and its unfavorable interactions with the defective insulin signaling, which in turn results in excessive accumulation of toxic lipid intermediates, potentially resulting in cardiac lipotoxicity that further impairs cardiac contractile functions (Chong et al., 2017). Aside from these well-characterized metabolic features in the stressed hearts, there has also been progress on the redox interplay of metabolic regulation in cardiac glucose and fatty acid metabolism. Cardiac-specific overexpression of NADPH oxidase 4 (Nox4) has recently been found to modulate substantial reprogramming of glucose and fatty acid metabolism in the heart of Nox4 transgenic (TG) mice compared to the wild type (WT) group (Nabeebaccus et al., 2017). Specifically, unstressed Nox4 TG hearts exhibited significantly decreased glucose oxidation rates and lowered glycolysis, subsequently increasing the accumulation of proximal glycolytic metabolites and upregulating fatty acid oxidation rate. Similar changes were observed in hypertrophied Nox4 TG hearts compared to the hypertrophied WT controls. Nox4 overexpression significantly promoted glucose diversion into the hexosamine biosynthetic pathway (HBP), producing the substrate, uridine diphosphate-*N*-acetylglucosamine (UDP-GlcNAc), for the posttranslational *O*-linked glycosylation (*O*-GlcNAcylation), via increasing activating transcription factor 4 and its direct transcriptional target, fructose-6-phosphate aminotransferase 1. While Nox4 TG and WT mice exhibited similar cardiac energetics and function at baseline, the former showed a significantly preserved energetic state when challenged with acute isoproterenol compared with WT control. Together, these results suggested that Nox4-redirected glucose utilization and subsequently increased fatty acid oxidation might be a beneficial response of the myocardium adapting to stress.

Previously, most studies investigating cardiac fatty acid and glucose metabolism were performed in an *ex vivo* model of perfused, isolated hearts. In recent years, the rapid development and application of isotopic labeling and metabolomics have greatly encouraged and facilitated the assessment of fuel availability and fate in the heart *in vivo*. For example, Schnelle et al. (2020) assessed the metabolic fate of glucose in the hearts of mice that underwent 2 weeks of transverse aortic constriction (TAC) and aortocaval fistula (shunt) surgery, respectively, via *in vivo* [U-^13^C] glucose labeling combined with isotopomer analysis with nuclear magnetic resonance spectroscopy (NMR spectroscopy). Male adult mice received 30-min continuous intravenous infusion of [U-^13^C] glucose before their hearts were excised for the *ex vivo* analysis. In pressure overloaded TAC hearts that exhibited significant concentric hypertrophy and reduced EF, ^13^C was significantly enriched in lactate, succinate, glutamate, glutamine, and aspartate in the heart compared with the sham group but were overall less pronounced in the plasma. There were no significant changes of ^13^C enrichment between volume overloaded shunt-treated hearts that showed prominent eccentric hypertrophy with unchanged LVEF and the sham group. However, increased plasma ^13^C enrichment of succinate was detected in the shunt group while the abundance of aspartate was too low in the plasma to trace its ^13^C enrichment. These findings indicated that their experimental approach primarily captured the cardiac-driven metabolic flux of glucose instead of systemic effects. The enrichment of [1,2,3-^13^C] lactate metabolized from [1,2,3-^13^C] pyruvate following glycolysis was significantly increased in TAC hearts together with markedly upregulated expressions of glucose transporter 1 (GLUT1), hexokinase-1, and lactate dehydrogenase A (LDHA) compared with the sham group. Both the enrichment of [4,5-^13^C] glutamate as a readout for the activity of pyruvate dehydrogenase (PDH) and [2,3-^13^C] glutamate indicative of anaplerotic reactions in the TCA cycle was significantly increased in TAC hearts compared with sham together with increased gene expression of PDH E1 component subunit α (PDHαE1), which suggested augmented pyruvate flux directly into the TCA cycle and greater anaplerosis. Such changes were not observed in the shunt group. However, the gene expression of pyruvate dehydrogenase kinase 4 (PDK4) was markedly decreased compared with the sham group. In addition, other than in shunt, TAC hearts exhibited a significant increase in the enrichment of [4,5-^13^C] glutamine and the total levels of glutamine measured by NMR as well as the protein levels of glutamine synthetase and glutaminase compared with sham. The *in vivo* ^13^C-labeling strategy employed in this study held unique advantages of directly tracing the metabolic pathway activities over conventional studies in which pathway analysis relied heavily on detecting changes in gene, mRNA, and protein expression levels that indirectly reflected pathway activities. The results gained also corroborated with the previously observed general pattern of increased glucose metabolism in pressure overload-induced heart failure.

In addition, Fulghum et al. (2022) recently examined the impact of cardiac-specific alterations of phosphofructokinase-1 (PFK1) activity on ancillary biosynthetic pathways of glucose metabolism in the myocardium of adult male mice by tracing the metabolic fate of dietary ^13^C_6_-glucose administered through a standardized ^13^C_6_-containing liquid diet. By overexpressing cardiac phosphatase-deficient or kinase-deficient 6-phosphofructo-2-kinase/fructose-2,6-bisphosphatase (PFK-2/FBPase-2/PFKFB), the Glyco*^Lo^* and Glyco*^Hi^* hearts were excised and subjected to NMR and ion exchange chromatography-mass spectrometry (IC-MS) to qualify metabolite abundance and ^13^C enrichment. In this study, Glyco*^Lo^* mice hearts with low PFK-1 activity exhibited increased glycogen synthetic metabolites such as glucose-6-phosphate (G6P) and fructose-6-phosphate, and glucose-1-phosphate together with increased ^13^C-labeled glycogen (Fulghum et al., 2022). However, cardiac PFK-1 activity did not significantly affect the HBP pathway but mildly enhanced the levels of metabolic intermediates involved in pyrimidine biosynthetic pathways. Moreover, both unlabeled and ^13^C-labeled 5-aminoimidazole-4-carboxamide-1-β-D-ribofuranoside (AICAR) and phosphoribosyl pyrophosphate were markedly increased in the Glyco*^Lo^* hearts. Further investigation revealed that the metabolic routing of glucose-derived ^13^C carbons to AICAR induced by low PFK-1 activity was associated with chaperones-containing large multimeric complexes and metabolic enzymes, such as phosphoribosylaminoimidazole carboxylase. This study focused on the relatively slower biosynthetic pathways of glucose metabolism compared with the majority of *in vivo* ^13^C labeling approaches previously reported (Josan et al., 2013; Schnelle et al., 2020), in which ^13^C glucose was administered by infusion or injections and thus was limited to only tracing the fast turnover pathways such as glycolysis due to their transient nature. Besides, these methods could not only induce stress responses because of involved procedures such as anesthesia or physical restraint but also could produce unknown artifacts that could cause potential discrepancies, which could be eliminated by oral administration.

### Ketone Bodies

A recent study demonstrated that ketone bodies could serve as a primary fuel source for cardiac energy production instead of a “thrifty-fuel” as high 3-OHB concentrations in aerobically perfused hearts extracted from 12-week-old male mice could markedly boost ketone oxidation and efficiently produce ATP without suppressing physiological glucose and palmitate metabolism rates (Ho et al., 2021). This finding collaborated with the lately recognized theory that ketones are the “preferred” fuel for the heart and the brain, despite the presence of adequate glucose. Their study did not observe improved cardiac efficiency upon increased ketone oxidation in healthy normal mice models. On the contrary, the diminished capacity of the failing heart to utilize fatty acids and glucose drives the metabolic machinery to shift to the alternative ketone oxidation pathway, raising circulating ketone bodies and promoting D-β-hydroxybutyrate dehydrogenase (BDH1) activities that catalyze ketone bodies back to acetyl coenzyme A (acetyl-CoA) (Aubert et al., 2016). Permanently elevated plasma ketone levels by knocking out the 3-oxoacid CoA-transferase 1 gene that encodes the ketolytic succinyl-CoA:3-ketoacid-CoA transferase 1 could ameliorate inflammatory infiltrates in failing hearts of male mice at 8–10 weeks of age subjected to TAC (Byrne et al., 2020). Therefore, while ketone bodies seem to be just a super fuel for a healthy heart, they have been further explored as a therapeutic strategy, such as acute intravenous infusion of 3-OHB to provide an additional fuel source to maintain energy stability in the failing myocardium (Lopaschuk et al., 2020). Uchihashi et al. (2017) produced a cardiac-specific Bdh1 overexpression TG mouse model to study ketone body metabolism in pressure overloaded hearts. Increased ketone body oxidation was observed in the hearts of both Bdh1 transgenic male mice at 10–12 weeks of age and sex-and age-matched mice who were subjected to TAC-induced pressure overload. *In vitro*, adenovirus-mediated Bdh1 overexpression was found to markedly mitigate 3-OHB-induced intracellular reactive oxygen species (ROS) production and cardiomyocyte apoptosis while enhancing the mRNA expressions of antioxidant enzymes such as metallothionein 2, catalase, and superoxide dismutases in H9c2 cells that were exposed to 5-mmol/L 3-OHB. Besides, the magnitude of 3-OHB-induced increase in histone hyperacetylation was greater in Bdh1 TG mice than WT control treated with the same infusion. The novel Bdh1 TG model provided further evidence of intervening myocardial ketone body metabolism as a potential therapeutic strategy for treating heart failure.

### Lactate

The myocardium has been considered a net lactate consumer that uptakes lactate directly proportional to its circulating concentrations over the past few decades (Gertz et al., 1988; Gibb and Hill, 2018). During high-intensity exercises, serum lactate is massively elevated under fully aerobic conditions, which outweighs glucose oxidation and starts to dominate the fuel supply for the heart via the cell–cell lactate shuttle that carries lactate from the skeleton muscles to the myocardium (Brooks, 2021). During uncompensated MI, circulating lactate levels also surges due to poor systemic perfusion, which has been clinically correlated as an independent prognostic marker for acute MI patients (Frydland et al., 2019). Aside from being a crucial cardiac fuel and major gluconeogenic precursor, transient lactate accumulation from anaerobic glycolysis also acts as a ‘ physicochemical buffer’ that could blunt acute aggravation of the ischemic injury at early reperfusion in myocardial ischemia/reperfusion injury (Qiao et al., 2013).

### Amino Acids

Amino acids play an obligatory role in maintaining the metabolic milieu of the heart, thus gaining more and more attention for exerting a marked influence on cardiac pathophysiology. Amino acids, such as glutamine and glutamate, can replenish a small amount of ATP in the stressed heart via non-oxidative metabolism, thus free from contributing to acidification and free radical formation (Drake et al., 2012). Glutamine is the most abundant amino acid in the body, and the majority of it is degraded into glutamate by glutaminase (glutaminolysis), which is consumed by the heart more than any other amino acid. Thus, glutamine and glutamate are considered preferential fuels for cardiac metabolism and have been highlighted as anaplerotic precursors of TCA intermediates. Watanabe et al. (2021) conducted an isotope tracing study to assess metabolic remodeling regarding the intracellular levels of glutamine, glutamate, and α-ketoglutarate in rat neonatal cardiomyocytes subjected to H_2_O_2_-induced oxidative stress. H_2_O_2_-treated cardiomyocytes exhibited enhanced glutaminolysis and a subsequent increase in ATP production through glutamine anaplerotic reactions to compensate for compromised substrate oxidation under immense oxidative stress, thus conferring cardioprotective effects in the stressed heart.

In recent years, there has also been some rapid progress on branched-chain amino acids (BCAAs) metabolism involved in cardiac metabolic remodeling, and it is receiving more attention nowadays than ever before. BCAAs, referring to leucine, isoleucine, and valine, serves as a major nitrogen donor for glutamine synthesis in muscles where BCAA catabolism primarily occurs due to robust BCAA transaminase (BCAT) activity that converts BCAAs and α-KG to α-ketoacids (BCKA) and glutamate. Defective BCAA catabolism and subsequent tissue accumulation of toxic byproducts such as medium- and long-chain acylcarnitines (AC), valine catabolite, 3-hydroxyisobutyrate (3HIB) as well as BCAA themselves have been extensively reported to impair fasting glucose and stimulate mammalian target of rapamycin complex 1(mTORC1) overactivation-induced insulin resistance (IR) (Yoon, 2016; Bloomgarden, 2018).

Sun et al. (2016) studied a knockout mouse model with deficient expression of protein phosphatase 2C in mitochondria (PP2Cm) and resultant inhibition on branched-chain alpha-keto acid dehydrogenase (BCKD) activity. Intramyocardial BCKA levels were significantly elevated in the heart of PP2Cm-KO mice after fasting, reaching the BCKA levels shown in failing WT mouse and human hearts recruited in this study. This increase became much greater in PP2Cm-KO hearts from fed mice, suggesting a potential influence of diet on cardiac BCAA accumulation. Functionally, 3-month-old PP2Cm-KO mice exhibited a modest decrease in systolic cardiac function, which was further reduced in 18-month-old PP2Cm-KO mice compared with age-matched WT. Since there were no major cardiac remodeling events in young PP2Cm-KO hearts, such a finding suggested that abnormal BCAA catabolism alone could induce systolic dysfunction over time without any external pathological stimuli. Moreover, PP2Cm-KO mice subjected to 8-week pressure overload started to exhibit signs of heart failure, highlighting an increased susceptibility to heart failure under pathological stress.

Dietary BCAAs have been repeatedly observed to have a damaging effect on the stressed heart (Wang et al., 2016; Li et al., 2020; Jiang et al., 2021). However, there is still a lack of sufficient knowledge about how dietary BCAA impacts cardiac metabolic dynamics and its interaction with cardiac function. Latimer et al. (2021) interrogated whether the timing of BCAA consumption could impact cardiometabolic outcomes in healthy and pressure-overloaded hearts as both temporal and spatial substrate abundance and partitioning could determine the metabolic fate of the substrates. For starters, mice at 20 weeks of age fed with a 4 h-long late high BCAA diet (LHB) at Zeitgeber time (ZT) 16 of the dark (active) phase exhibited a significant drop in cardiac output but a rapid and dramatic increase in both biventricular weight and cardiomyocyte size compared with those on a late low BCAA diet (LLB), whereas no hypertrophic responses were observed in mice fed with an early high BCAA diet (EHB) at ZT12. Upon further probing, cardiac protein synthesis was found to be dramatically elevated in LHB mice compared with LLB mice over BCAA selectively promoted activation of the mTOR signaling at the end of the active period. However, although mice subjected to a 4-week LHB diet exhibited hypertrophic responses at ZT24 compared with EHB mice, there were no significant cardiac changes at ZT16 between LHB mice and EHB mice, revealing a transient and highly dynamic nature of BCAA-induced cardiac growth. Additionally, cardiac-specific BMAL1 knockout (CBK) mice that displayed a dysfunctional circadian clock were recruited and fed with a high BCAA diet either at ZT12 or ZT16 for 4 weeks using the long-term EHB and LHB feeding regimens. There were no significant differences in cardiac mass, cardiomyocyte size, or mTOR activity between EHB and LHB fed CBK mice, suggesting that circadian misalignment could abolish time-of-day dependent cardiac responsiveness of dietary BCAAs. Moreover, CBK mice fed with a normal BCAA diet in an *ad libitum* manner exhibited significant cardiac hypertrophic responses compared with littermate control, whereas oscillatory fluctuations in mTORC1 activities observed in control littermates were completely abolished in CBK mice, indicating that clock-defective mice chronically developed increased sensitivity to a BCAA diet. Given that BCAA-induced hypertrophic growth in the healthy mouse hearts did not establish an accumulative effect, mice were subjected to TAC surgery to induce cardiac pressure overload and were fed with either the LHB diet or EHB diet. LHB mice exhibited significant hypertrophy and fibrosis and declined function compared with EHB mice, implying that repetitive BCAA consumption at the end of the active phase could build up a damaging effect on the pressure-overloaded heart. Overall, this study was the first to probe how circadian rhythms converged with BCAA metabolism to modulate cardiac remodeling, which recapitulated the importance of the often-neglected temporal regulation of cardiac metabolites in both the normal and stressed heart.

As a prevalent yet understudied BCAA intermediate, the metabolic fate of BCKA has been surrounded with inquisitive eyes and was recently traced to be primarily reanimated back to valine and subsequent catabolite, 3-hydroxyisobutyrate in a BCAT-dependent manner in the heart of recruited male rats (Walejko et al., 2021). Furthermore, accumulation of reaminated BCAA together with persistently increased BCKA supply in BCKA-perfused hearts of obese mice could activate total protein synthesis by promoting phosphorylation of eukaryotic translation initiation factor 4E-binding protein 1, thus revealing a potential pathophysiological mechanism of cardiac BCKA reamination in obesity-induced cardiac metabolic remodeling.

Despite these exciting discoveries, it is notable that BCAAs accounted for less than 5% of total carbon combustion in the heart; thus, defective BCAA catabolism observed in heart failure is likely to be mediated by alternative mechanisms beyond the control of energy production.

### Comprehensive Regulation and Dynamic Adaptation of Nutrient Utilization in the Heart

In addition to the above experiment studies using animal models primarily focused on characterizing each substrate utilization and the underlying molecular mechanisms in the heart individually, offering only a partial insight into the whole picture of dynamic adaptation of nutrient utilization in the heart, many recent studies focus on the comprehensive regulation among different metabolic substrates and the dynamic adaptation of nutrient utilization in the heart.

Newhardt et al. (2019) tested the impact of constitutively enhanced cardiac glycolysis on cardiac metabolism and mitochondrial function in response to nutrient availability using a transgenic mouse model (Glyco*^Hi^* mice) that expressed sustained glycolysis through the increased activity of phosphofructokinase-2 (PFK-2). Both transgenic mice and their WT littermates were fed with either a standard low-fat chow diet (LFD) or challenged with a 7-day high-fat diet (HFD). As a result, both the genotype and the diet manipulation significantly affected global cardiac metabolism due to increased depletion of early glycolytic intermediates by upregulated glycolysis. Subsequent global proteomic analysis revealed an increased abundance of proteins involved in both glucose and lipid metabolism in HFD-treated Glyco*^Hi^* hearts, whereas HFD-treated WT mice had consistently greater abundance in lipid metabolism proteins but declined abundance in proteins involved in glucose metabolism, suggesting that Glyco*^Hi^* mouse hearts were able to sustain elevated expression of glycolytic proteins against short-term HFD challenge. There was also a striking increase of BCAAs in HFD-treated Glyco*^Hi^* mice, accompanied by a diet-independent decrease in BCKD E1α subunit. Such an increase in BCAAs was considered a unique effect as other amino acids had a decreased abundance in Glyco*^Hi^* hearts compared to WT. This study depicted both genotype and diet-dependent impact of sustained glycolysis on global cardiac metabolism, in which PFK-2 induced increase in the content of BCAAs and PDK4 was identified as novel compensatory mechanisms of the heart to manage nutrient stress. This study also provides clues to potential outcomes of metabolic remodeling that could occur if glucose metabolism takes up a larger proportion of cardiac energy metabolism in a healthy heart.

Fillmore et al. (2018) subjected 12-weeks old mice to HFD and LFD, respectively, for 10 weeks to directly examine the relationship between cardiac BCAA oxidation and IR. In this study, the isolated, perfused hearts from mice with HFD-induced IR exhibited significantly reduced BCAA oxidation rate, markedly impaired glucose oxidation and the ability of insulin to promote glucose oxidation compared with the LFD group. The results clarified that inhibited BCAA oxidation negatively impacted cardiac insulin signaling potentially through increasing BCAA accumulation rather than inhibiting fatty acid and glucose oxidation. Uddin et al. (2021) recruited a cardiac-specific mitochondrial BCAT (BCATm) knockout mice model to investigate whether it was BCAAs or BCKAs or both of them that was incriminated in the mediation of IR. 8-week-old BCATm^–/–^ male mice exhibited decreased BCAA oxidation, increased individual BCAAs, and decreased BCKAs in the heart, with circulating BCAA levels remaining unchanged compared with their control counterparts. These results indicated that increased accumulation of BCAAs instead of BCKAs should be held responsible for triggering the mTOR-mediated hypertrophic responses in the heart, and BCKAs could exert an inhibitory effect on insulin-promoted cardiac glucose oxidation potentially through downregulating AKT and PDH activities.

Additionally, Li et al. (2017) demonstrated the novel metabolic and physiological functions of BCAA catabolism in the heart by investigating cardiac metabolic flux in PP2Cm-KO mice 2–3 months of age. PP2CmKO mice exhibited elevated circulating and cardiac BCAA levels as expected compared with WT littermates accompanied by a significant inhibition on glycogen synthesis from exogenous glucose, which was further attributed to suppressed glucose uptake following selectively reduced mitochondrial pyruvate flux. Further investigation revealed that chronic BCAAs accumulation at high concentrations could substantially inhibit PDH activity by suppressing *O*-GlcNAcylation. Although there were no evident pathological changes at baseline, KO mice subjected to myocardial IR insults exhibited worsened I/R injury compared with WT littermates when administered with a high level of BCAA supplements. Such negative changes could be rescued by either 3,6-dichlorobenzo[b]thiophene-2-carboxylic acid (BT2) treatment or GLUT1 overexpression in double transgenic PP2CmKO/GLUT1TG mice. Li’s study revealed a novel regulatory role of BCAA in cardiac glucose metabolism and an increased vulnerability of the PP2CmKO heart to IR injury following defective BCAA catabolism.

Reciprocally, cardiac glucose metabolism was found to negatively regulate BCAA breakdown through the actions of the above-described transcriptional regulator, Kruppel like factor 15 (KLF15) (Shao et al., 2018). RNA microarray and gene ontology enrichment analysis on transgenic mouse hearts (Glut1-TG) at the age of 10–12 weeks with cardiac-specific GLUT1 overexpression revealed that downregulated genes within the BCAA degradation pathway were significantly enriched, especially KLF15, along with dramatically decreased expression of key BCAA catabolic enzymes, particularly BCAT2, BCKDH, and PP2Cm. Applied metabolomics detected a higher level of glycolytic metabolites, a selective increase in BCAAs, and reduced downstream BCAA catabolites following enhanced glucose uptake in Glut1-TG hearts, which suggested that glucose could negatively modulate BCAA breakdown. Further investigation on KLF15 regulation of BCAA metabolism identified downregulated binding of the cAMP response element-binding protein to the KLF15 promoter region in cultured cardiomyocytes exposed to high glucose or overexpressing Glut1, indicating that glucose served as a negative regulator of KLF15, through which BCAA degradation was held back. This study then returned to play with pathological cardiac hypertrophy, suggesting a potential association between glucose reliance and BCAA degradation in pathological cardiac hypertrophy. This study further uncovered that the glucose-KLF15-BCAA degradation pathway enabled cardiac hypertrophic growth under pathological stimuli through the actions of its end-effector mTOR, whose sustained activation required glucose to down-regulate BCAA degradation. The above two studies combined revealed a negative reciprocal regulatory axis between BCAA and glucose metabolism in the normal heart, as well as how metabolic rewiring in the context of cardiac ischemia and pressure overload stealthily induced pathological cardiac remodeling.

Altogether, these studies have advanced our understanding of how fatty acid and glucose metabolism regulates BCAA and contributes to the normal physiology of the heart as well as how alterations in BCAA catabolism impact the well-characterized derangements in glucose and lipid metabolism intensifies maladaptive metabolic remodeling in the stressed heart. This emerging new paradigm implicating defective metabolic regulation in the pathogenesis of major adverse cardiac events could provide essential knowledge for developing efficacious therapeutic strategies such as pharmacologically inhibiting BCKD activity with BT2 treatment to restore BCAA catabolic flux in pressure-overload induced heart failure and myocardial IR injury (Li et al., 2017; Chen et al., 2019).

However, the fundamental structure supporting cardiac metabolic plasticity and flexibility is, no doubt, the mitochondria. Whether it is lipids, polysaccharides, or proteins, they are all degraded into acetyl-CoA and then enter the TCA cycle coupled with oxidative phosphorylation apparatus inside the mitochondria to generate ATP. Therefore, the mitochondria serve as a converging hub for energy substrate crossovers and play a central role in metabolic homeostasis. Dysfunctional mitochondria are directly responsible for remodeled metabolic patterns and disruption of energy homeostasis in the heart, which underlie the pathological outcomes of many heart diseases. For example, deficient mitochondrial pyruvate carrier 1, which transports pyruvate from glycolysis into the mitochondria, could lead to ventricular hypertrophy, heart failure, and premature death in mice hearts upon a series of metabolic remodeling, predominantly glucose overreliance and diminished TCA activity (Zhang et al., 2020). Meanwhile, although this paper primarily focused on major metabolic substrates in the heart, it is no doubt that all the metabolic intermediates fulfilled a crucial and unique role in cardiac metabolism that requires further profiling and identification. All the discussed studies are summarized in Table 1.

**TABLE 1:** Summarization of recent studies on myocardial substrate utilization involved in cardiac pathophysiology.

Major metabolic substrates studied in the heart	Research focus and highlights
Fatty acid and glucose	Characterization of myocardial glucose metabolism in heart failure (Chong et al., 2017; Nabben et al., 2018).
	Deep metabolic network tracing *in vivo*; stable isotope tracing; ^13^C metabolic flux analysis of glucose metabolism (Schnelle et al., 2020).
Ketone	Ketone utilization as a preferred fuel in the heart of healthy normal mice (Ho et al., 2021).
	Cardiac protection of OXCT1 knockout in TAC-induced heart failure (Byrne et al., 2020).
	BHB treatment in the failing myocardium as an additional fuel source (Lopaschuk et al., 2020).
	Cardioprotection of Bdh1 overexpression in TAC-induced heart failure (Uchihashi et al., 2017).
Lactate	Lactate dominates myocardial fuel supply during exercises (Brooks, 2021).
Glutamate and glutamine	Isotope tracing study on cardioprotection from myocardial oxidative stress and major fuel deficit through anaplerotic reactions (Watanabe et al., 2021).
BCAAs	Cardiac BCAA accumulation impairs insulin signaling (Fillmore et al., 2018).
	Further identification of differential roles of BCAAs and BCKAs in the heart using BCATm knockout model (Uddin et al., 2021).
	Molecular mechanisms of BCAA catabolism in PP2Cm deficient heart (Sun et al., 2016).
	Negative regulation of defective cardiac BCAA catabolism on glucose metabolism in PP2Cm deficient heart (Li et al., 2017).
	Identification of glucose-KLF15-BCAA degradation pathway in cardiac BCAA catabolism (Shao et al., 2018).
	Temporal regulation of BCAA metabolism in the heart (Latimer et al., 2021).
	The metabolic fate of BCKA (Walejko et al., 2021).

*PFK-1, phosphofructokinase-1; Nox4, NADPH oxidase 4; OXCT1, 3-oxoacid CoA-transferase 1; BHB, beta-hydroxybutyrate; Bdh1, 3-hydroxybutyrate dehydrogenase 1; MI, myocardial infarction; BCAA, branched-chain amino acid; BCKAs, branched-chain α-keto acids; BCATm, mitochondrial branched-chain aminotransferase; PP2Cm, protein phosphatase 2Cm; KLF15, Kruppel Like Factor 15.*

## Metabolic Adaption in the Heart Under Physiological Conditions

Although metabolic remodeling predominantly occurs in the cardiac pathological condition in response to mechanical stress, myocardial metabolism can also adapt to the energy demand under the different physiological conditions. While the cardiac metabolism remodeling in pathological conditions has been well-reviewed (Wang et al., 2014; Chen et al., 2019; Geraets et al., 2019; Tran and Wang, 2019), much fewer studies have been on the metabolic adaption in the heart under physiological conditions despite the importance of the topic. Thus, we summarized the recent discovery in physiologic cardiac metabolic modification, focusing on exercise and cardiac development.

### Exercise-Induced Metabolic Remodeling in the Heart

Exercises have been reported to induce cardiomyocyte hypertrophy, promote physiological cardiac growth, and improve cardiovascular health. The metabolic responses ensuing different types of exercises vary, shaping the heart muscle broadly into either eccentric or concentric hypertrophy with individual heterogeneity (Weeks and McMullen, 2011). Endurance training, usually referred to as aerobic exercises, such as swimming, walking, or jogging on a treadmill, tends to elicit eccentric hypertrophy along with chamber dilation due to greater growth in myocyte length than width, whereas resistance training or anaerobic exercises such as free weights tends to induce concentric hypertrophy without chamber dilation resulted from a greater increase in myocyte width over length (Fernandes et al., 2011). Heart-pumping aerobic exercises, or in other terms, cardiovascular exercises, could progressively challenge the cardiorespiratory system and improve their function and performance, thus benefiting many aspects of health, especially heart health.

So far, how exercise-induced adaptations in cardiac metabolism promote cardiac remodeling has not yet been fully investigated. First of all, metabolic flexibility of the mitochondria as an efficient adaptation to energy resources plays a pivotal role in balancing periodic shifts between primary fuel utilization such as glucose and FA oxidation as well as employing alternative fuels upon dynamic energy demand and supply in the heart. How endurance exercises promote cardiac mitochondrial structural and functional quality has been extensively reported in cardiac disease models, particularly in diabetic and failing hearts (Campos et al., 2017; Wang et al., 2020). For example, in diabetic mouse hearts, treadmill exercise could markedly improve cardiac function by mitigating oxidative stress from accumulated ROS inside the mitochondria while boosting its oxidative capacity by increasing oxidative enzyme activities. Not only that, endurance training by treadmill could switch myocardial metabolism from the defective fatty acid oxidation that undermines cardiac efficiency to glucose oxidation through restoring the cardiac expression of peroxisome proliferator-activated receptor-γ coactivator (PGC-1α).

In non-stressed hearts of 8-week-old male mice, endurance exercise was reported to induce mitochondrial biogenesis through the actions of nitric oxide, a key mediator of cardiovascular responses to exercises (Vettor et al., 2014). Compared with the hearts of sedentary WT mice, swim-trained WT mouse hearts exhibited markedly increased endothelial nitric oxide synthase (eNOS) mRNA, protein, and activity accompanied by increased mitochondrial DNA (mtDNA) content and higher mRNA levels of PGC-1α, nuclear respiratory factor 1, and mitochondrial transcription factor A, which are master regulators of mitochondrial biogenesis. However, swim training failed to induce mitochondrial biogenesis nor subsequent increase in cardiomyocyte glucose uptake in eNOS knockout (eNOS^–/–^) mice compared to sedentary (eNOS^–/–^) mice. In contrast, trained WT mice exhibited significantly increased cardiac GLUT1 expression and upregulated insulin sensitivity that markedly promoted cardiac glucose uptake, all of which remained unchanged in trained eNOS^–/–^ mice. These findings revealed novel eNOS-dependent mitochondrial biogenesis induced by endurance exercise, which substantially orchestrated metabolic adaptations of the heart to exercise through promoting basal and insulin-stimulated cardiac glucose uptake.

Besides, Sun et al. (2019) demonstrated exercise-induced insulin-independent glucose transport in treadmill-trained male mice at 8–12 weeks old through the action of slightly but significantly increased Thr208 phosphorylation of sucrose non-fermenting AMPK-related kinase (SNARK), a crucial regulator of glucose uptake previously characterized in skeletal muscle. Explicitly, SNARK overexpression could markedly increase basal instead of insulin-stimulated glycogen content in WT SNARK-transfected HL1 cardiomyocytes compared with empty vector (EV) cells, whereas exercise-stimulated basal glucose transport was completely blunted in both SNARK (shRNA)-infected HL1 cells and the heart of trained SNARK heterozygotic knockout mice compared with control cells and WT littermates, respectively, while insulin-stimulated glucose transport remained essentially unchanged. Taken together, the above studies demonstrated that exercise-induced basal and insulin-dependent glucose transport and uptake serve as a crucial chapter for exercise-promoted metabolic remodeling in the heart, providing a valid therapeutic perspective in improving the capability of cardiomyocytes to take up and utilize glucose. Following its uptake, glucose enters the glycolytic pathway upon being phosphorylated by hexokinase to G6P. Glycolysis is tightly controlled by the coordinated actions of glycolytic enzymes, which without doubt pose an imperative impact on downstream glucose oxidation in the heart.

Gibb et al. (2017) also characterized regular exercise-induced metabolic periodicity on glucose metabolism through glycolytic activity modulation. Trained male and female mice were discovered to exhibit 15% higher circulating insulin-like growth factor I (IGF-1) than sedentary mice. Next, the bifunctional 6-phosphofructo-2-kinase/fructose-2,6-biphosphatase 2 (PFKFB2) that catalyzes F2,6BP synthesis and breakdown in the heart was examined for its phosphorylation level at S483. Compared to sedentary mice, significantly increased PFKFB2 phosphorylation at S483 by Akt in response to insulin and IGF-1 was detected in the exercise-adapted heart of trained mice, potentially contributing to upregulated glycolytic activities in the exercise-adapted heart. In contrast, during early recovery or right after a bout of exercise, PFK-2 phosphorylation was acutely diminished, accompanied by evident glycogen buildup and a sharp fall in circulating glucose, suggesting substantially reduced glucose utilization which eventually returned to an elevated level during full recovery. Furthermore, cardiac-specific kinase-deficient (Glyco*^Lo^*) and phosphatase-deficient (Glyco*^Hi^*) TG mice were developed to constitutively manipulate myocardial PFK-1 activity, which resulted in mild cardiac hypertrophy in both Glyco*^Lo^* and Glyco*^Hi^* mice accompanied by irreversible mitochondrial inflexibility. Only Glyco*^Lo^* hearts exhibited seemingly beneficial cardiac remodeling patterns similar to but not exceeding that of exercise-adapted WT mice and without evident upstream AKT activation. Additionally, Newhardt et al. (2019) studied the separate and combined influence of metabolic genotypes and diet types on cardiac metabolism in Glyco*^Hi^* hearts that expressed sustained glycolysis through increased PFK-2 activity. Both Glyco*^Hi^* mice and their WT littermates at 30–36 weeks of age were fed with either a standard LFD or challenged with a 7-day HFD. As a result, both the genotype and the diet manipulation significantly affected global cardiac metabolism. Pathway analysis uncovered a marked increase of PDK4 in HFD-treated WT hearts, and such increase was even more pronounced in HFD-treated Glyco*^Hi^* mouse hearts reflected through PDH activity. Besides, fasted Glyco*^Hi^* mouse hearts exhibited significantly higher PDK4 levels than fasted WT hearts, and fed Glyco*^Hi^* mouse hearts also had greater PDK4 levels than fed WT mouse hearts, suggesting that enhanced glycolysis could elicit an increase in PDK4 to dial down glucose oxidation. The above two studies together provided unique evidence for the pervasive effects of sustained glycolysis on cardiac metabolic remodeling at the mechanistic level in response to exercises and nutrient availability, respectively, which further contributed to an improved understanding of dynamic interactions between diet, exercises, and genotypes, the three major components necessary to determine individual cardiac health.

More than that, myocardial expression of CCAAT enhancer binding protein beta (Cebpb), whose reduction has previously been reported to serve as a central signal controlling exercise-induced physiological hypertrophy (Boström et al., 2010), was observed to remain unchanged between exercise-adapted and sedentary Glyco*^Lo^* mice. Meanwhile, myocardial expression of Cited4, whose cardiac overexpression has been shown to induce physiological hypertrophy with normal systolic function that mimics the effects of endurance exercises (Bezzerides et al., 2016), was found to bear no statistically significant difference between the two groups. On the contrary, WT mice subjected to treadmill training exhibited significantly downregulated myocardial Cebpb expression and upregulated Cbp/P300 interacting transactivator with Glu/Asp rich carboxy-terminal domain 4 (Cited4) expression, which was consistent with the previously reported findings compared with sedentary mice. Besides, Glyco*^Lo^* hearts exhibited intrinsically lower myocardial Cebpb expression and higher Cited4 expression at baseline than their WT counterparts. These results together suggest that constitutively low glycolysis alone was enough for maximal cardiac growth through modulating the Cebpb/Cited4 transcriptional program, whereas Glyco*^Hi^* hearts developed a more dilated phenotype and did not engage in gene programs that induce physiological cardiac growth. Additionally, both Glyco*^Lo^* and Glyco*^Hi^* hearts exhibited substantially altered myocardial gene expressions involved in various cellular processes and signaling compared with WT hearts. In particular, among an array of differentially regulated genes by glycolysis, low glycolytic rates in Glyco*^Lo^* hearts exerted a strong influence on genes involved in redox reactions, lipid metabolism, and cell responses to cAMP, whereas high levels of glycolysis in Glyco*^Hi^* hearts had a major impact on genes involved in cellular responses to cAMP and hormone stimuli, angiogenesis and differentiation, as well as transcriptional activities. Collectively, this study serves as a paradigmatic example in demonstrating how exercise-induced dynamic changes in cardiac metabolites modulate cardiac remodeling within and beyond the realm of fuel metabolism.

Davogustto et al. (2021) investigated the impact of mTORC1 in the development of cardiac hypertrophy of the adult mice subjected to voluntary exercise training, whose sustained activation was unraveled to modulate cardiac glucose metabolism preceding a subsequent hypertrophic response. In the said study, a transgenic mouse model with inducible, cardiac-specific sustained mTORC1 activation through tuberous sclerosis complex 2 deletion (mTORC1*^iSA^*) developed a concentric LV hypertrophy accompanied with increased systolic function at 28-week old compared to littermate controls, which resembled adaptive cardiac hypertrophy. Retrogradely perfused mTORC1*^iSA^* heart at 12-week old exhibited markedly decreased glucose uptake and oxidation while the downstream product of hexokinase 2, G6P, was significantly increased, suggesting a considerably decreased glucose influx to the glycolytic pathway. Further investigation discovered a persistent decrease in protein expression and enzymatic activity of glucose 6-phosphate isomerase (GPI) from as early as 12-week old all the way to 28-week old. WT mice subjected to 6-day wheel running showed increased mTOR phosphorylation at Ser 2448 accompanied by decreased GPI protein expression but increased enzymatic activities compared with sedentary WT mice, whereas oral administration of rapamycin for the last 8 weeks of the project prevented the increase in GPI activity at 12-week old and hypertrophic alterations seen at 28-week old in both mTORC *^iSA^* mice and trained WT mice. WT mice subjected to a 6-week wheel running protocol developed significantly increased cardiac hypertrophic changes together with increased GPI activity, which were again prevented with the rapamycin diet, suggesting a consistent rapamycin inhibition on mTORC1-induced glucose changes during both short-term and long-term voluntary exercises. The mTORC1 signaling has been considered as a metabolic rheostat that integrates inputs from nutrient sensing with cell growth (Valvezan and Manning, 2019). Sustained mTORC1 activation has been reported to play an adaptive role in both physiological and pathological hypertrophic hearts, whose inhibition has frequently been described to confer cardioprotection in the stressed heart (Daneshgar et al., 2021). The non-pathological cardiac metabolic changes and subsequent hypertrophic responses observed in this study revealed a continuum hypertrophic outcome rather than the generally accepted dichotomous pattern between physiological and pathologic cardiac remodeling under the mediation of mTORC1. Meanwhile, such a study recapitulated the stealth metabolic remodeling processes that preceded, triggered, and sustained structural and functional remodeling of the heart to a measurable extent.

**FIGURE 1:**
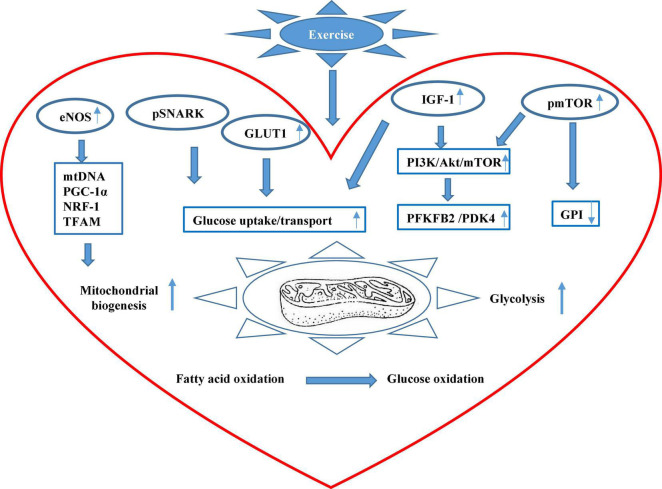
The scheme of major mechanisms of exercise-promoted metabolic remodeling in the heart summarized in this review. Exercise can improve cardiac mitochondrial biogenesis through the eNOS signaling pathway that promotes mitochondrial DNA (mtDNA) content, peroxisome proliferator-activated receptor γ coactivator 1α (PGC-1α), nuclear respiratory factor 1 (NRF-1), and mitochondrial transcription factor A (TFAM). Exercise can also upregulate the phosphorylation level of sucrose non-fermenting AMPK-related kinase (SNARK), enhancing glucose uptake and utilization. Besides, exercise stimulates circulating insulin-like growth factor (IGF-1) and insulin that increase overall glucose transport and uptake and also upregulates serine phosphorylation of 6-phosphofructo-2-kinase/fructose-2,6-biphosphatase 2 (PFKFB2) that boost glycolysis in the heart. In addition, exercise augments the activation of the mammalian target of rapamycin (mTOR) and reduces the activity of glucose 6-phosphate isomerase (GPI). Systemically, exercise can stimulate peripheral fatty acid mobilization from adipose tissue, and increased palmitoleic acid (C16:1n7) stimulates Akt phosphorylation that mediates metabolic remodeling in the heart.

Following the fast-acting glucose that quickly gets depleted during exercise, fatty acid oxidation takes the place of glucose oxidation to keep up with the increased energy demand in the heart accompanied by increased lipolytic activities in adipose tissue. Thus, alterations in fatty acid metabolism could naturally serve as a sensitive indicator that predicts exercise-driven cardiac metabolic shift. Foryst-Ludwig et al. (2015) provided novel evidence that palmitoleic acid (C16:1n7), a common fatty acid constituent of white adipose tissue (WAT) triglycerides (TG), played an indispensable role in prolonged physical exercise-induced non-proliferative left ventricle hypertrophy LVH. Since adipose triglyceride lipase (Atgl) catalyzes the initial step in adipose TG lipolysis, adipose tissue-specific Atgl knockout (atATGL-KO) female mice at 5-week-old age with accordingly curtailed WAT lipolysis were subjected to chronic treadmill running together with their WT littermates. As expected, trained WT mice exhibited decreased cardiac glucose uptake and considerably increased cardiac fatty acid uptake along with significantly increased LV mass compared to sedentary WT mice. Reciprocally, genetic deletion of Atgl expression in trained atATGL-KO mice completely abrogated exercise-induced cardiac fatty acid uptake, resulting in an attenuated cardiac hypertrophic response, which revealed that FAs potentially served as prohypertrophic mediators. Intriguingly, C16:1n7 was singled out from a mixture of fatty acids for exerting prohypertrophic effects in cultured HL-1 cells was significantly increased in trained WT mice, but not atATGL-KO mice. Furthermore, short-term treatment of HL-1 cells with C16:1n7 greatly augmented Akt phosphorylation, and dietary C16:1n7 enrichment successfully salvaged the impaired LVH induced by exercise in trained atATGL-KO mice, bringing it to a comparable level with their WT littermates under sedentary conditions. Serum FA analysis on highly trained endurance human athletes revealed a markedly significant linear correlation between C16:1n7 level and diastolic interventricular septum thickness, suggesting a therapeutic potential using lipid-derived supplements to prevent adverse cardiovascular events. The findings from the above study combined provided a fresh perspective that peripheral substrate metabolism such as FA mobilization from adipose tissues did not just provide energy supply upon substrate partitioning in the heart but also took part in the exercise-induced metabolic and physiological remodeling processes of the heart. This study also reminded us that we should not only concentrate on the heart itself when investigating alterations in cardiac metabolism but extend further into the mutual interplay between cardiac and systemic metabolism. The main findings from these new studies have been summarized in Figure 1.

### Metabolic Remodeling in Heart Development

Another type of physiological metabolic remodeling in the heart occurs during normal cardiac development. Difference from the adult heart, the fetal heart mainly utilizes glucose and lactate as predominant sources to generate ATP due to carbohydrate-enriched nutrient supply. Immediately after birth, the neonatal heart quickly switches to fatty acid oxidation upon mitochondrial reorganization, at least partially driven by dramatically increased oxygen availability and increased workload while maintaining its glycolytic capacity (Lemay et al., 2021). The unique metabolic transitions in the heart during cardiac maturation have been intensively studied but are still not fully elucidated. Studying fetal metabolic adaptations in the heart contributes to a better understanding of the pathogenic mechanisms in adult heart diseases, such as ischemia, hypertrophy, and heart failure. In heart failure, impaired mitochondria oxidative capacity drives a stereotypic metabolic shift from primary fatty acid oxidation to glucose oxidation accompanied by reactivation of several fetal genes whose presence is believed to attune the failing myocardium to non-physiological hypoxia and preserve cardiac function till a breakpoint (van der Pol et al., 2020).

Hypoxia-inducible factor (HIF)-1 has been a primary focus during the fetal metabolic transition whose deficiency has been reported to markedly attenuate cardiomyogenesis of embryonic stem cells in mice (Kudová et al., 2016). Highly expressed HIF provides embryonic cardiomyocytes sufficient resistance to the hypoxic intrauterine environment and meanwhile promotes cardiac anaerobic glycolysis through upregulating a suite of glycolytic genes (Cerychova and Pavlinkova, 2018). In contrast, HIF expression drastically declines after birth allowing a fatty-acid dominant to quickly occur, which powers a subsequent adaptive hypertrophic response that profoundly drives cardiomyocytes to gradually lose their proliferative capacity and achieve terminal maturation. Breckenridge et al. (2013) reported that HIFα-signaling dependent cardiac transcription factor the heart and neural crest-derived transcript-1 (Hand1) that was highly expressed in the developing heart underwent a sharp fall right after birth, whereas its prolonged perinatal expression in Hand 1 TG mouse hearts was found to yield glycogen stores to depletion and meantime inhibit lipid uptake and incorporation into metabolic handling of fatty acid species compared with littermate controls potentially attributable to Hand1 overexpression-prevented increase in expression of genes involved in lipid metabolism (Breckenridge et al., 2013). This study identified a novel regulatory mechanism of the HIF1a/Hand1 signaling pathway in controlling cardiac fetal-neonatal energy switch, and experimentally upregulated Hand1 expression in adult hearts subjected to IR injury was observed to dramatically decrease cardiomyocyte death, suggesting its therapeutic potential in ischemia protection of the adult heart.

In addition, Walejko et al. (2018) adopted a multi-omics approach to integrating data from metabolomics, lipidomics, and transcriptomics in order to comprehensively profile fetal-neonatal metabolic transition in ovine heart tissues. The metabolomics analysis revealed a clear distinction of metabolite compositions between the term fetal heart and the neonatal heart. Metabolites, such as lactate, succinate, and alanine, were detected in greater abundance in fetal heart tissues, while AMP, glutamate, choline, and 1,2-propanediol were highly elevated in the neonatal heart. Lipid profiling of cardiac tissue disclosed a marked separation in lipids between fetal and neonatal hearts. Eight lipid classes showed greater abundance in neonatal cardiac tissues, of which TG accounted for the majority, whereas 7 lipid classes were found to be more elevated in fetal cardiac tissues, such as sphingomyelin, suggesting significantly altered lipid profile between fetal and neonatal cardiac tissues. Transcriptomic profiling revealed 893 significantly elevated genes in the neonatal heart and 940 significantly elevated genes in the fetal heart compared with each other. The differential expression of these genes was identified to regulate downstream pathways involved in BCAA degradation, fatty acid degradation, and the metabolic fate of β-alanine, butanoate, and tryptophan. Meanwhile, genes associated with cardiomyopathy were also shown to be strongly expressed in the fetal heart, which corresponded to hypertrophic remodeling of the term fetal heart during terminal maturation. Further integrated analysis of the above omics together unraveled multiple clusters of cardiac metabolic pathways of lipids, amino acids, and glucose, respectively, which were consistent with the altered expression of genes controlling the corresponding metabolic activities. This multi-omics approach adequately captured the dynamic metabolic alterations during heart maturation, in which highly expressed transcripts for genes involved in BCAA degradation pathways in the fetal heart offered additional insight into a potential mechanism by which increased BCAA catabolism could promote cardiac protein synthesis and growth in the term fetal heart.

A transcriptome analysis conducted by Iruretagoyena et al. (2014) profiled metabolic genes in the developing heart using human fetal cardiac tissues prospectively collected between 10 and 18 weeks of gestational age, which were divided into three groups as 10–12 (H1), 13–15 (H2), and 16–18 (H3) weeks. Their study detected significantly increased gene expressions involved in either fatty acid metabolism or structural remodeling in H3 hearts compared with H1 hearts, while glycolytic genes remained highly but not differentially expressed in all three groups. Their results were highly consistent with previous animal studies on fetal heart maturation that fatty acid metabolism started to dominate as the fetus developed while glucose oxidation was consistently maintained at a high level to provide basal energy production in the fetal heart.

So far, various animal species have been recruited to study fetal heart growth, such as the murine and ovine fetal hearts described above. However, considering distinct interspecies differences in embryo development and limited samples of human fetal heart, *in vitro* human embryonic stem cell-derived cardiomyocytes (hESC-CMs) with an immature metabolic phenotype have been extensively used to study human fetal cardiomyocyte metabolic maturation and potentially provide alternative cell-based approaches to cardiac regeneration and repair (Liu et al., 2018; Romagnuolo et al., 2019). Nakano et al. (2017) investigated specifically how changes in glucose metabolism manipulated hESC-CMs maturation by culturing them in media containing various concentrations of glucose. Their study observed that hESC-CMs cultured in low glucose media exhibited more robust differentiation and a consistent increase in cardiomyocyte size compared with those cultured in high glucose. Moreover, glucose deprivation was found to boost mitochondrial respiration in hESC-CMs, promoting its functional maturation, whereas high glucose conditions dose-dependently blocked mitochondrial pyruvate utilization in hESC-CMs and greatly held back cardiac maturation through a metabolic pathway parallel to glycolysis, the pentose phosphate pathway (PPP) whose oxidative branch generates precursors for nucleotide biosynthesis. Further, uridine rescue was observed to substantially restore the reduced proliferative capacity of hESC-CMs cultured in low glucose, while nucleotides deprivation, on the contrary, markedly induced hESC-CMs maturation following glucose deprivation, suggesting that the inhibitory effects of glucose on hESC-CMs maturation depended on nucleotide synthesis through the PPP pathway. Subsequent *in vivo* study revealed drastically downregulated fetal cardiac glucose uptake albeit relatively stable uterine glucose supply during late gestation or the early postnatal period. Additionally, fetal and neonatal hearts from diabetic pregnancy of Akita heterozygous mice showed elevated mitotic activity and increased biventricular wall thickness in company with decreased cardiomyocyte size compared with control littermates, suggesting that hyperglycemia could impede fetal cardiac maturation and potentially contribute to cardiomyopathy in infants of diabetic mothers (Nakano et al., 2017). This study provides novel evidence on glucose metabolism from the PPP pathway instead of the glycolytic pathway mediated the balance between proliferation and differentiation of fetal cardiomyocytes through nucleotide biosynthesis.

Hu et al. (2018) studied hESC-CMs cultured in media with different compositions, specifically glucose-containing media, glucose media with the addition of fatty acids, and glucose-free fatty acids only media, to investigate potential signaling pathways underpinning the metabolic and functional maturation of hESC-CMs. Glucose cultured hESC-CMs exhibited significantly higher hexokinase activities and lactate levels than those cultured in media with fatty acids alone, which indicated a glycolytic dominant metabolic regulation mimicking embryonic and postnatal cardiac metabolic patterns. Contrarily, hESC-CMs cultured in media with fatty acids alone displayed greatly enhanced mitochondrial oxidative capacity and ATP production compared with those cultured in media containing either glucose or glucose and fatty acids, featuring adult-like cardiac metabolic characteristics. Further exploration revealed aberrant activation of HIF1α with higher nuclear localization and subsequently increased LDHA expression in glucose cultured hESC-CMs compared with those cultured in fatty acid medium lacking sugar source. Conversely, inhibition of the HIF1α/LDHA axis was discovered to promote metabolic maturation of hESC-CMs through attenuated glycolysis and enhanced oxidative respiration to generate more ATP. The above studies on *in vitro* hESC-CMs metabolic maturation well corroborated with *in vivo* discoveries regarding fetal cardiac metabolic changes during terminal maturation, leading to a more comprehensive understanding of fetal cardiac metabolic remodeling.

Emanuelli et al. (2021) very recently characterized the metabolic profile of human cardiomyocytes induced from pluripotent stem cell-derived cardiomyocytes (hiPSC-CM) during their maturation from 6 to 12 weeks in culture. hiPSC-CMs exhibited ongoing structural maturation but no significant change in the glycolytic reserve, glucose uptake, or main glycolytic enzymes over 6 weeks of prolonged culture. The expression of PDHαE1 was increased twofold together with an evident isoform switch between pyruvate dehydrogenase kinase-1 (PDK1) and PDK4 at week 12, whereas ^13^C isotope labeling revealed that glucose flux branching into the PPP pathway was significantly lowered at week 12. Besides, although there was no significant difference in overall protein *O*-GlcNAcylation, both UDP-GlcNAc abundance and glutamine-fructose-6-phosphate aminotransferase 2 expression were significantly reduced at week 12, suggesting reduced flux to the HBP pathway. These findings indicated that the metabolic flux that passed through the glycolytic pathway was preferentially directed toward the entry of the TCA cycle. hiPSC-CM at week 12 also exhibited a more mature mitochondrial network and a tendency to shift to a more oxidative metabolic profile over time as fatty acid oxidation increasingly contributed to total oxygen consumption rate at the cost of glucose oxidation.

**FIGURE 2:**
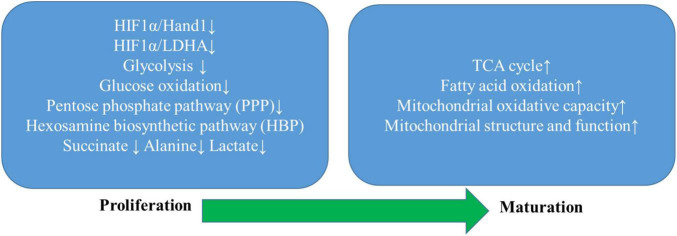
Summarization of progress of metabolic remodeling in the developing heart. The developing heart undergoes a unique metabolic switch from the primary utilization of glucose through the glycolytic pathway and auxiliary biosynthetic pathways, including the pentose phosphate pathway (PPP) and the hexosamine biosynthesis pathway (HBP) to fatty acid oxidation as the mitochondrial structure and function mature to substantially improve mitochondrial oxidative capacity. Intermediate metabolites that are specifically increased during proliferation, such as lactate, alanine, succinate, and triglycerides, are markedly decreased during maturation. At the transcriptional level, maturation of cardiomyocytes relies on a sharp decrease in the expression of the hypoxia-inducible factor 1 subunit a (HIF1a), HIF1a-dependent cardiac transcription factor heart, and neural crest-derived transcript-1 (Hand1), and lactate dehydrogenase A (LDHA). Thus, inhibition of the HIF1α/Hand1 and HIF1α/LDHA signaling plays a crucial role in the time-dependent metabolic shift toward increased metabolic flux to the TCA cycle as the predominant metabolic pathway of oxidative metabolism.

Overall speaking, the fetal metabolic transition is achieved through coordinated control and regulation of substrate partitioning under the combinatorial effects of the metabolic and signaling network, especially the repeatedly highlighted HIF1α signaling both *in vivo* and *in vitro*. In addition, studying the progression of fetal cardiac remodeling conferred substantial value on deciphering the pathological metabolic alterations in the ischemic and failing hearts, thus encouraging the development of minimally invasive and regenerative therapeutics for a series of heart diseases. The main findings from these new studies have been summarized in Figure 2.

## Conclusion and Future Perspectives

This review primarily focused on presenting the current knowledge of myocardial substrate metabolism and the latest progress regarding cardiac metabolic remodeling under physiological conditions such as during exercise and cardiac development. Especially, many novel signaling pathways have been linked to the metabolic remodeling in the heart under physiological conditions of endurance exercises and cardiac development, which remarkably increases our knowledge of the heart’s potential in response to the states with diverse energy requirements under the physiological condition. We also dabbled into the correlation of metabolic patterns between physiological and pathological cardiac metabolism, which all together strived to gain a deeper understanding of the impact of metabolic remodeling in the heart under physiological conditions. Despite the significant impacts of these advances in this field, many aspects remain unknown and require future investigations:

### New Insights in Substrate Utilization and Associated Signaling in Metabolic Remodeling

It is no doubt that every substrate matters, and each substrate serves not only as a fuel source but also as an indispensable part of the metabolic signaling pathways that together induce structural and functional remodeling in the heart. The current studies bring new insights into the substrate utilization of the heart in physiological conditions. First, although glucose and fatty acid metabolism remain a research hotspot, cardiac lactate and ketone metabolism have gained growing attention, especially the ketone body, whose increase serves as a general feature of maladaptive metabolism in the aging heart and the failing heart. As a result, a large body of research has been devoted to investigating their pervasive cardioprotective effects by applying a ketogenic diet or administering exogenous ketogenic agents to induce therapeutic ketosis. Secondly, many emerging roles of BCAA metabolism in the heart have been brought to light, such as the relationship between cardiac BCAA oxidation and insulin resistance; transcriptional regulation of BACC catabolism by upstream regulators such as KFL15, BCAT2, BCKDH, and PP2Cm; third, most importantly, as previous studies primarily focused on characterizing each substrate utilization and the underlying molecular mechanisms in the heart individually, many recent studies focus on the interaction among different metabolic substrates and the dynamic adaptation of nutrient utilization in the heart, by using a combination of the various animal models, such as the newly discovered metabolic interplay and reciprocal regulation between glucose and BCAA metabolism. These studies highlighted the comprehensive regulations in cardiac metabolism.

Despite the exciting findings from the recent studies, it remains largely unknown regarding the complex regulatory mechanisms governing cardiac energy substrate metabolism under physiological conditions, not to mention numerous unidentified metabolic remodeling processes under pathological stimuli. For example, although physiological and pathological myocardial hypertrophy has long been considered as two separate entities and sustained, mTORC1 activation was heavily incriminated in producing divergent hypertrophic outcomes in them, the recently reported intermediate non-pathological-hypertrophic phenotype in the presence of persistent mTORC1 activation indicative of a continuum of influence of this key regulator on cardiac growth. Additionally, glycolytic activities were observed to exhibit a periodic pattern in exercise-adapted hearts, and genetically downregulated cardiac glycolysis could maximize physiological myocardial hypertrophy. The more newly discovered metabolic pathways aside from the most characterized IGF-1 and mTOR signalings involved in exercise-promoted cardioprotection, such as the eNOS and the SNARK signaling, greatly contributed to identifying novel promising molecular targets. Furthermore, the differential role of BCAAs and BCKAs on mediating cardiac IR has been recently clarified, and recruiting BCAA oxidation as a promising treatment target to improve insulin sensitivity in heart diseases associated with IR has become a new focus. So far, only a few genes and transcriptional factors involved in BCAA metabolism were identified and analyzed as shown above, which says that very little is known about the underpinning pathways and molecular regulation of BCAA metabolism in response to various physiological and pathological stimuli as well as its potential interactions with other metabolic processes in the heart, which require further investigations in the future. It is beyond doubt that cardiac research on metabolism is heading toward the path of multidisciplinary analysis and quantification with a plethora of powerful and promising molecular methods that can more accurately and comprehensively characterize cardiac metabolism.

### New Approaches for Measuring Cardiac Metabolism

The conventional *ex vivo* and *in vitro* models and experimentation exerted more uncertainty in interpreting the gained results to the *in vivo* system. On top of these traditional techniques, the application of novel strategies to enable deep metabolic network tracing or studies employing advanced analytical technologies and informatics to unravel the dynamic and complex metabolic nature of cardiomyocytes. Systemic delivery of metabolic substrates, such as C16:1n7, could exert a localized effect on exercise-induced cardiac growth, which served as a fresh reminder that metabolic remodeling under any circumstance is an outcome of both local and systemic metabolic responses. The advancement of metabolomics and isotope tracing as well as their individual and combined applications in studying heart metabolism *ex vivo* and *in vivo* has provided novel and alternative approaches for characterizing cardiac metabolism. So far, it remains challenging to investigate cardiac metabolic changes in response to exercise training due to its dynamic nature of systemic involvement, thus causing larger magnitudes of more complex discrepancies. Therefore, the application of metabolomics, isotope tracing, and other future technological innovations will substantially provide more accuracy and reduce the difficulties in delineating numerous exercise-promoted cardiac metabolic signatures. In addition, alternative experimental approaches such as *in vitro* hESC-CMs and hiPSC-CMs testing revealed more knowledge of the metabolic changes during fetal cardiomyocyte proliferation and maturation. Studying metabolic remodeling in the developing heart also brought promising therapeutic targets as fetal metabolic gene reprogramming has been repeatedly recognized in the adult heart under pathological conditions.

### Therapeutic Potential

Recent studies also provide evidence for developing new therapeutic strategies. For example, most recent studies have indicated that dietary BCAAs could be used as a potentially efficacious intervention for heart failure in some reports. However, in others, they have been shown to increase the susceptibility of the heart to pathological stimuli. The conflict on this topic remains unsolved as the specific experimental models and diet protocols employed in each study varied, and the hemostasis of BCAA metabolism in the heart is always the result of multifactorial regulation, such as the often understudied temporal regulations. There are a significant number of targets for potential therapeutic interventions waiting to be discovered within the metabolic realm, in which pharmacological targeting of energy metabolic signaling and pathways has just emerged. Therefore, our quest has just begun to uncover the entire blueprint of cardiac metabolism.

## Author Contributions

XS performed the literature searching and drafted the manuscript. HQ revised the manuscript. Both authors contributed to the article and approved the submitted version.

## Conflict of Interest

The authors declare that the research was conducted in the absence of any commercial or financial relationships that could be construed as a potential conflict of interest.

## Publisher’s Note

All claims expressed in this article are solely those of the authors and do not necessarily represent those of their affiliated organizations, or those of the publisher, the editors and the reviewers. Any product that may be evaluated in this article, or claim that may be made by its manufacturer, is not guaranteed or endorsed by the publisher.

## References

[B1] Ahmad T. Kelly J. P. Mcgarrah R. W. Hellkamp A. S. Fiuzat M. Testani J. M. (2016). Prognostic Implications of Long-Chain Acylcarnitines in Heart Failure and Reversibility With Mechanical Circulatory Support. *J. Am. Coll. Cardiol.* 67 291–299. 10.1016/j.jacc.2015.10.079 26796394 PMC5429585

[B2] Aubert G. Martin O. J. Horton J. L. Lai L. Vega R. B. Leone T. C. (2016). The Failing Heart Relies on Ketone Bodies as a Fuel. *Circulation* 133 698–705. 10.1161/circulationaha.115.017355 26819376 PMC4766035

[B3] Bezzerides V. J. Platt C. Lerchenmüller C. Paruchuri K. Oh N. L. Xiao C. (2016). CITED4 induces physiologic hypertrophy and promotes functional recovery after ischemic injury. *JCI Insight* 1:e85904. 10.1172/jci.insight.85904 27430023 PMC4945110

[B4] Bloomgarden Z. (2018). Diabetes and branched-chain amino acids: what is the link?. *J. Diabetes* 10 350–352. 29369529 10.1111/1753-0407.12645

[B5] Boström P. Mann N. Wu J. Quintero P. A. Plovie E. R. Panáková D. (2010). C/EBPβ controls exercise-induced cardiac growth and protects against pathological cardiac remodeling. *Cell* 143 1072–1083. 10.1016/j.cell.2010.11.036 21183071 PMC3035164

[B6] Breckenridge R. A. Piotrowska I. Ng K. E. Ragan T. J. West J. A. Kotecha S. (2013). Hypoxic regulation of hand1 controls the fetal-neonatal switch in cardiac metabolism. *PLoS Biol.* 11:e1001666. 10.1371/journal.pbio.1001666 24086110 PMC3782421

[B7] Brooks G. A. (2021). Role of the Heart in Lactate Shuttling. *Front. Nutr.* 8:663560. 10.3389/fnut.2021.663560 33968972 PMC8101701

[B8] Byrne N. J. Soni S. Takahara S. Ferdaoussi M. Al Batran R. Darwesh A. M. (2020). Chronically Elevating Circulating Ketones Can Reduce Cardiac Inflammation and Blunt the Development of Heart Failure. *Circ. Heart Fail.* 13:e006573. 10.1161/CIRCHEARTFAILURE.119.006573 32493060

[B9] Campos J. C. Queliconi B. B. Bozi L. H. M. Bechara L. R. G. Dourado P. M. M. Andres A. M. (2017). Exercise reestablishes autophagic flux and mitochondrial quality control in heart failure. *Autophagy* 13 1304–1317. 10.1080/15548627.2017.1325062 28598232 PMC5584854

[B10] Cerychova R. Pavlinkova G. (2018). HIF-1, Metabolism, and Diabetes in the Embryonic and Adult Heart. *Front. Endocrinol.* 9:460. 10.3389/fendo.2018.00460 30158902 PMC6104135

[B11] Chen L. Song J. Hu S. (2019). Metabolic remodeling of substrate utilization during heart failure progression. *Heart Fail. Rev.* 24 143–154. 10.1007/s10741-018-9713-0 29789980

[B12] Chong C. R. Clarke K. Levelt E. (2017). Metabolic Remodeling in Diabetic Cardiomyopathy. *Cardiovasc. Res.* 113 422–430. 10.1093/cvr/cvx018 28177068 PMC5412022

[B13] Daneshgar N. Rabinovitch P. S. Dai D. F. (2021). TOR Signaling Pathway in Cardiac Aging and Heart Failure. *Biomolecules* 11:168. 10.3390/biom11020168 33513917 PMC7911348

[B14] Davogustto G. E. Salazar R. L. Vasquez H. G. Karlstaedt A. Dillon W. P. Guthrie P. H. (2021). Metabolic remodeling precedes mTORC1-mediated cardiac hypertrophy. *J. Mol. Cell Cardiol.* 158 115–127. 10.1016/j.yjmcc.2021.05.016 34081952 PMC8630806

[B15] Drake K. J. Sidorov V. Y. Mcguinness O. P. Wasserman D. H. Wikswo J. P. (2012). Amino acids as metabolic substrates during cardiac ischemia. *Exp. Biol. Med.* 237 1369–1378. 10.1258/ebm.2012.012025 23354395 PMC3816490

[B16] Emanuelli G. Zoccarato A. Reumiller C. M. Papadopoulos A. Chong M. Rebs S. (2021). A roadmap for the characterization of energy metabolism in human cardiomyocytes derived from induced pluripotent stem cells. *J. Mol. Cell Cardiol.* 164 136–147. 10.1016/j.yjmcc.2021.12.001 34923199

[B17] Fernandes T. Soci U. P. Oliveira E. M. (2011). Eccentric and concentric cardiac hypertrophy induced by exercise training: microRNAs and molecular determinants. *Braz. J. Med. Biol. Res.* 44 836–847. 10.1590/s0100-879x2011007500112 21881810

[B18] Fillmore N. Wagg C. S. Zhang L. Fukushima A. Lopaschuk G. D. (2018). Cardiac branched-chain amino acid oxidation is reduced during insulin resistance in the heart. *Am. J. Physiol. Endocrinol. Metab.* 315 E1046–E1052. 10.1152/ajpendo.00097.2018 30106622

[B19] Foryst-Ludwig A. Kreissl M. C. Benz V. Brix S. Smeir E. Ban Z. (2015). Adipose Tissue Lipolysis Promotes Exercise-induced Cardiac Hypertrophy Involving the Lipokine C16:1n7-Palmitoleate. *J. Biol. Chem.* 290 23603–23615. 10.1074/jbc.M115.645341 26260790 PMC4583014

[B20] Frydland M. Møller J. E. Wiberg S. Lindholm M. G. Hansen R. Henriques J. P. S. (2019). Lactate is a Prognostic Factor in Patients Admitted With Suspected ST-Elevation Myocardial Infarction. *Shock* 51 321–327. 10.1097/SHK.0000000000001191 30286032

[B21] Fulghum K. L. Audam T. N. Lorkiewicz P. K. Zheng Y. Merchant M. Cummins T. D. (2022). In vivo deep network tracing reveals phosphofructokinase-mediated coordination of biosynthetic pathway activity in the myocardium. *J. Mol. Cell Cardiol.* 162 32–42. 10.1016/j.yjmcc.2021.08.013 34487754 PMC8766935

[B22] Geraets I. M. E. Glatz J. F. C. Luiken J. Nabben M. (2019). Pivotal role of membrane substrate transporters on the metabolic alterations in the pressure-overloaded heart. *Cardiovasc. Res.* 115 1000–1012. 10.1093/cvr/cvz060 30938418

[B23] Gertz E. W. Wisneski J. A. Stanley W. C. Neese R. A. (1988). Myocardial substrate utilization during exercise in humans. Dual carbon-labeled carbohydrate isotope experiments. *J. Clin. Invest.* 82 2017–2025. 10.1172/JCI113822 3198763 PMC442784

[B24] Gibb A. A. Epstein P. N. Uchida S. Zheng Y. Mcnally L. A. Obal D. (2017). Exercise-Induced Changes in Glucose Metabolism Promote Physiological Cardiac Growth. *Circulation* 136 2144–2157. 10.1161/CIRCULATIONAHA.117.028274 28860122 PMC5704654

[B25] Gibb A. A. Hill B. G. (2018). Metabolic Coordination of Physiological and Pathological Cardiac Remodeling. *Circ. Res.* 123 107–128. 10.1161/CIRCRESAHA.118.312017 29929976 PMC6023588

[B26] Ho K. L. Karwi Q. G. Wagg C. Zhang L. Vo K. Altamimi T. (2021). Ketones can become the major fuel source for the heart but do not increase cardiac efficiency. *Cardiovasc. Res.* 117 1178–1187. 10.1093/cvr/cvaa143 32402081 PMC7982999

[B27] Hu D. Linders A. Yamak A. Correia C. Kijlstra J. D. Garakani A. (2018). Metabolic Maturation of Human Pluripotent Stem Cell-Derived Cardiomyocytes by Inhibition of HIF1α and LDHA. *Circ. Res.* 123 1066–1079. 10.1161/CIRCRESAHA.118.313249 30355156 PMC6208155

[B28] Iruretagoyena J. I. Davis W. Bird C. Olsen J. Radue R. Teo Broman A. (2014). Metabolic gene profile in early human fetal heart development. *Mol. Hum. Reprod.* 20 690–700. 10.1093/molehr/gau026 24674993 PMC11514182

[B29] Jiang Y. J. Sun S. J. Cao W. X. Lan X. T. Ni M. Fu H. (2021). Excessive ROS production and enhanced autophagy contribute to myocardial injury induced by branched-chain amino acids: roles for the AMPK-ULK1 signaling pathway and α7nAChR. *Biochim. Biophys. Acta Mol. Basis Dis.* 1867:165980. 10.1016/j.bbadis.2020.165980 32980459

[B30] Josan S. Park J. M. Hurd R. Yen Y. F. Pfefferbaum A. Spielman D. (2013). In vivo investigation of cardiac metabolism in the rat using MRS of hyperpolarized [1-13C] and [2-13C]pyruvate. *NMR Biomed.* 26 1680–1687. 10.1002/nbm.3003 23904148 PMC3838505

[B31] Kudová J. Procházková J. Vašièek O. Pereèko T. Sedláèková M. Pešl M. (2016). HIF-1alpha Deficiency Attenuates the Cardiomyogenesis of Mouse Embryonic Stem Cells. *PLoS One* 11:e0158358. 10.1371/journal.pone.0158358 27355368 PMC4927095

[B32] Latimer M. N. Sonkar R. Mia S. Frayne I. R. Carter K. J. Johnson C. A. (2021). Branched chain amino acids selectively promote cardiac growth at the end of the awake period. *J. Mol. Cell Cardiol.* 157 31–44. 10.1016/j.yjmcc.2021.04.005 33894212 PMC8319101

[B33] Lemay S. E. Awada C. Shimauchi T. Wu W. H. Bonnet S. Provencher S. (2021). Fetal Gene Reactivation in Pulmonary Arterial Hypertension: GOOD, BAD, or BOTH?. *Cells* 10:1473. 10.3390/cells10061473 34208388 PMC8231250

[B34] Li T. Zhang Z. Kolwicz S. C. Jr. Abell L. Roe N. D. Kim M. (2017). Defective Branched-Chain Amino Acid Catabolism Disrupts Glucose Metabolism and Sensitizes the Heart to Ischemia-Reperfusion Injury. *Cell Metab.* 25 374–385. 10.1016/j.cmet.2016.11.005 28178567 PMC5301464

[B35] Li Y. Xiong Z. Yan W. Gao E. Cheng H. Wu G. (2020). Branched chain amino acids exacerbate myocardial ischemia/reperfusion vulnerability via enhancing GCN2/ATF6/PPAR-α pathway-dependent fatty acid oxidation. *Theranostics* 10 5623–5640. 10.7150/thno.44836 32373236 PMC7196282

[B36] Liu Y. W. Chen B. Yang X. Fugate J. A. Kalucki F. A. Futakuchi-Tsuchida A. (2018). Human embryonic stem cell-derived cardiomyocytes restore function in infarcted hearts of non-human primates. *Nat. Biotechnol.* 36 597–605. 29969440 10.1038/nbt.4162 PMC6329375

[B37] Lopaschuk G. D. Karwi Q. G. Ho K. L. Pherwani S. Ketema E. B. (2020). Ketone metabolism in the failing heart. *Biochim. Biophys. Acta Mol. Cell Biol. Lipids* 1865:158813. 10.1016/j.bbalip.2020.158813 32920139

[B38] Murashige D. Jang C. Neinast M. Edwards J. J. Cowan A. Hyman M. C. (2020). Comprehensive quantification of fuel use by the failing and nonfailing human heart. *Science* 370 364–368. 10.1126/science.abc8861 33060364 PMC7871704

[B39] Nabben M. Luiken J. Glatz J. F. C. (2018). Metabolic remodelling in heart failure revisited. *Nat. Rev. Cardiol.* 15:780. 10.1038/s41569-018-0116-7 30367134

[B40] Nabeebaccus A. A. Zoccarato A. Hafstad A. D. Santos C. X. Aasum E. Brewer A. C. (2017). Nox4 reprograms cardiac substrate metabolism via protein O-GlcNAcylation to enhance stress adaptation. *JCI Insight* 2:e96184. 10.1172/jci.insight.96184 29263294 PMC5752273

[B41] Nakano H. Minami I. Braas D. Pappoe H. Wu X. Sagadevan A. (2017). Glucose inhibits cardiac muscle maturation through nucleotide biosynthesis. *Elife* 6:e29330. 10.7554/eLife.29330 29231167 PMC5726851

[B42] Newhardt M. F. Batushansky A. Matsuzaki S. Young Z. T. West M. Chin N. C. (2019). Enhancing cardiac glycolysis causes an increase in PDK4 content in response to short-term high-fat diet. *J. Biol. Chem.* 294 16831–16845. 10.1074/jbc.RA119.010371 31562244 PMC6851294

[B43] Qiao X. Xu J. Yang Q. J. Du Y. Lei S. Liu Z. H. (2013). Transient acidosis during early reperfusion attenuates myocardium ischemia reperfusion injury via PI3k-Akt-eNOS signaling pathway. *Oxid. Med. Cell Longev.* 2013:126083. 10.1155/2013/126083 24312696 PMC3839119

[B44] Romagnuolo R. Masoudpour H. Porta-Sánchez A. Qiang B. Barry J. Laskary A. (2019). Human Embryonic Stem Cell-Derived Cardiomyocytes Regenerate the Infarcted Pig Heart but Induce Ventricular Tachyarrhythmias. *Stem Cell Rep.* 12 967–981. 10.1016/j.stemcr.2019.04.005 31056479 PMC6524945

[B45] Schnelle M. Chong M. Zoccarato A. Elkenani M. Sawyer G. J. Hasenfuss G. (2020). In vivo [U-(13)C]glucose labeling to assess heart metabolism in murine models of pressure and volume overload. *Am. J. Physiol. Heart Circ. Physiol.* 319 H422–H431. 10.1152/ajpheart.00219.2020 32648823 PMC7473922

[B46] Shao D. Villet O. Zhang Z. Choi S. W. Yan J. Ritterhoff J. (2018). Glucose promotes cell growth by suppressing branched-chain amino acid degradation. *Nat. Commun.* 9:2935. 10.1038/s41467-018-05362-7 30050148 PMC6062555

[B47] Sun H. Olson K. C. Gao C. Prosdocimo D. A. Zhou M. Wang Z. (2016). Catabolic Defect of Branched-Chain Amino Acids Promotes Heart Failure. *Circulation* 133 2038–2049. 10.1161/CIRCULATIONAHA.115.020226 27059949 PMC4879058

[B48] Sun X. L. Lessard S. J. An D. Koh H. J. Esumi H. Hirshman M. F. (2019). Sucrose nonfermenting AMPK-related kinase (SNARK) regulates exercise-stimulated and ischemia-stimulated glucose transport in the heart. *J. Cell Biochem.* 120 685–696. 10.1002/jcb.27425 30256437 PMC6347018

[B49] Tran D. H. Wang Z. V. (2019). Glucose Metabolism in Cardiac Hypertrophy and Heart Failure. *J. Am. Heart Assoc.* 8:e012673. 10.1161/JAHA.119.012673 31185774 PMC6645632

[B50] Uchihashi M. Hoshino A. Okawa Y. Ariyoshi M. Kaimoto S. Tateishi S. (2017). Cardiac-Specific Bdh1 Overexpression Ameliorates Oxidative Stress and Cardiac Remodeling in pressure Overload-Induced Heart Failure. *Circ. Heart Fail.* 10:e004417. 10.1161/CIRCHEARTFAILURE.117.004417 29242353

[B51] Uddin G. M. Karwi Q. G. Pherwani S. Gopal K. Wagg C. S. Biswas D. (2021). Deletion of BCATm increases insulin-stimulated glucose oxidation in the heart. *Metabolism* 124:154871. 10.1016/j.metabol.2021.154871 34478752

[B52] Valvezan A. J. Manning B. D. (2019). Molecular logic of mTORC1 signalling as a metabolic rheostat. *Nat. Metab.* 1 321–333. 10.1038/s42255-019-0038-7 32694720 PMC12569966

[B53] van Bilsen M. Van Nieuwenhoven F. A. Van Der Vusse G. J. (2008). Metabolic remodelling of the failing heart: beneficial or detrimental?. *Cardiovasc. Res.* 81 420–428. 10.1093/cvr/cvn282 18854380

[B54] van der Pol A. Hoes M. F. De Boer R. A. Van Der Meer P. (2020). Cardiac foetal reprogramming: a tool to exploit novel treatment targets for the failing heart. *J. Intern. Med.* 288 491–506. 10.1111/joim.13094 32557939 PMC7687159

[B55] Vettor R. Valerio A. Ragni M. Trevellin E. Granzotto M. Olivieri M. (2014). Exercise training boosts eNOS-dependent mitochondrial biogenesis in mouse heart: role in adaptation of glucose metabolism. *Am. J. Physiol. Endocrinol. Metab.* 306 E519–E528. 10.1152/ajpendo.00617.2013 24381004

[B56] Walejko J. M. Christopher B. A. Crown S. B. Zhang G. F. Pickar-Oliver A. Yoneshiro T. (2021). Branched-chain α-ketoacids are preferentially reaminated and activate protein synthesis in the heart. *Nat. Commun.* 12:1680. 10.1038/s41467-021-21962-2 33723250 PMC7960706

[B57] Walejko J. M. Koelmel J. P. Garrett T. J. Edison A. S. Keller-Wood M. (2018). Multiomics approach reveals metabolic changes in the heart at birth. *Am. J. Physiol. Endocrinol. Metab.* 315 E1212–E1223. 10.1152/ajpendo.00297.2018 30300011 PMC6336953

[B58] Wang S. Y. Zhu S. Wu J. Zhang M. Xu Y. Xu W. (2020). Exercise enhances cardiac function by improving mitochondrial dysfunction and maintaining energy homoeostasis in the development of diabetic cardiomyopathy. *J. Mol. Med.* 98 245–261. 10.1007/s00109-019-01861-2 31897508

[B59] Wang W. Zhang F. Xia Y. Zhao S. Yan W. Wang H. (2016). Defective branched chain amino acid catabolism contributes to cardiac dysfunction and remodeling following myocardial infarction. *Am. J. Physiol. Heart Circ. Physiol.* 311 H1160–H1169. 10.1152/ajpheart.00114.2016 27542406

[B60] Wang Z. V. Li D. L. Hill J. A. (2014). Heart failure and loss of metabolic control. *J. Cardiovasc. Pharmacol.* 63 302–313. 10.1097/FJC.0000000000000054 24336014 PMC3980079

[B61] Watanabe K. Nagao M. Toh R. Irino Y. Shinohara M. Iino T. (2021). Critical role of glutamine metabolism in cardiomyocytes under oxidative stress. *Biochem. Biophys. Res. Commun.* 534 687–693. 10.1016/j.bbrc.2020.11.018 33213841

[B62] Weeks K. L. McMullen J. R. (2011). The athlete’s heart vs. the failing heart: can signaling explain the two distinct outcomes?. *Physiology* 26 97–105. 10.1152/physiol.00043.2010 21487028

[B63] Yoon M. S. (2016). The Emerging Role of Branched-Chain Amino Acids in Insulin Resistance and Metabolism. *Nutrients* 8:405. 10.3390/nu8070405 27376324 PMC4963881

[B64] Zhang Y. Taufalele P. V. Cochran J. D. Robillard-Frayne I. Marx J. M. Soto J. (2020). Mitochondrial pyruvate carriers are required for myocardial stress adaptation. *Nat. Metab.* 2 1248–1264. 10.1038/s42255-020-00288-1 33106689 PMC8015649

[B65] Zuurbier C. J. Bertrand L. Beauloye C. R. Andreadou I. Ruiz-Meana M. Jespersen N. R. (2020). Cardiac metabolism as a driver and therapeutic target of myocardial infarction. *J. Cell Mol. Med.* 24 5937–5954. 10.1111/jcmm.15180 32384583 PMC7294140

